# A Study on the Structure of Ideal-Based Non-Zero Divisor Graphs Associated with *Z*_*n*_

**DOI:** 10.12688/f1000research.172788.1

**Published:** 2026-01-09

**Authors:** Sameer Kadem, Ali Abd Aubad

**Affiliations:** 1 Department of Mathematics, University of Baghdad, Baghdad, Baghdad Governorate, Iraq; 2 Department of Mathematics, University of Baghdad, Baghdad, Baghdad Governorate, Iraq

**Keywords:** non-zero divisor graph, cut-set, connected, degree, topological graph indices, dominating number

## Abstract

**Background:**

The study of algebraic structures through graph-theoretic representations provides a powerful visual and combinatorial framework for analyzing ring-theoretic properties. The ideal-based non-zero divisor graph $\emptyset_{I}\left(Z_{n}\right)$ , constructed from the ring of integers modulo n with respect to a proper ideal I . This graph extends the classical zero-divisor graph framework and serves as a visual and structural invariant for analyzing ideal interactions in finite commutative rings.

**Methods:**

Using combinatorial graph theory and modular arithmetic, we analyze fundamental properties of $\emptyset_{I}\left(Z_{n}\right)$ . Vertex degrees, connectivity, and cut-sets are characterized using divisibility conditions and the Euler totient function ϕ(n) . The analysis distinguishes cases based on the parity and primality of n , as well as the generator of I . Topological indices, including the Zagreb and Randić indices, are formulated to quantify structural complexity.

**Results:**

We establish necessary and sufficient conditions for the connectivity of $\emptyset_{I}\left(Z_{n}\right)$ , proving it is connected for all n≥10 and any non-zero proper ideal I . For prime n∉{2,3} , the graph is shown to be complete. General formulas are provided for calculating vertex degrees based on gcd(x,d) where I=<d> . Furthermore, the structure and computation cut-sets are characterized for $Z_{p^{2}}\;$ and composite n=xy . Moreover, the domination number γ ( $\emptyset_{I}\left(Z_{n}\right)$ )=1 and girth gr ( $\emptyset_{I}\left(Z_{n}\right)$ )=3 is established for n≥10 . General expressions for Zagreb and Randić indices are derived, directly linking graph invariants to n and d.

**Conclusions:**

The graph $\emptyset_{I}\left(Z_{n}\right)$ serves as an effective combinatorial invariant for studying the interplay between ideals and zero-divisor structure in $\;Z_{n}$ . These results establish systematic connections between ring-theoretic properties and graph parameters, enabling both qualitative and quantitative analysis through connectivity, degree distributions, cut-sets, and topological indices.

## 1. Introduction

By transforming algebraic structures into graphs, the algebraic properties can be visualized, hidden symmetries can be uncovered, and intricate relationships can be simplified. By revealing patterns and invariants, graph theory tools facilitate the intuitive study of zero-divisors, ideals, and ring structures. This helps with classification, problem solving, and computational exploration by bridging the gap between abstract algebra and combinatorics. For an example, see Refs. 1– 7. For the commutative ring R and a proper ideal I of R , Redmond ^8^ was the first to identify the ideal-based zero divisor graph, designated as $\Gamma_{I}\left(R\right),$ which contains a vertex set $\{\alpha \;\in \;I^{C}:\alpha \beta \;\in \;I,$ for some $\beta \;\in \;I^{C}\}$ , and two distinct vertices μ and λ are adjacent if μλ ∈I . Inspired by the preceding definition, we construct a simple graph associated with a ring R and a proper ideal I of R , denoted as $\emptyset_{I}\left(R\right)$ , With the exception of the ideal I , the multiplicative identity, and its additive inverse, every vertex represents an element of R. Moreover, an edge connects two different vertices only when their product (in either order) is not in the ideal I . This type of graph is known as a non-zero divisor graph when I = 0; various studies have investigated this instance. ^9– 11^ In this study, we assume that all graphs are undirected and that, basic graphs are devoid of many edges or loops. A graph Γ is said to be connected if a path exists between every pair of vertices. The girth of the graph represents the length of the smallest cycle. When a graph does not have any cycles, its girth is considered as infinite. Subset D⊆V(Γ) is said to be the dominating set of a graph Γ if every vertex $\alpha \;\in \;D^{c}$ is adjacent to at least one vertex in D. The maximal cardinality of the smallest set that dominates G is represented by the domination number, which is abbreviated as γ(G). Additional definitions pertaining to graph theory can be found in Ref. 12. In this study, we look at certain essential characteristics of the $\emptyset_{I}\left(\mathrm{Zn}\right)$ graphs, illustrating their connectivity, and formulate general equations for the cut sets, vertex degrees, and specific topological degree-based indices. The results delineate cut-Sets, establish generic criteria for connectedness, and formulate expressions for vertex degrees based on divisibility relations in $\emptyset_{I}\left(\mathrm{Zn}\right)$ . Moreover, many topological indices have been computed to assess symmetry and structural complexity.

## 2. Preliminaries

This section first outlines the definition of the ideal-based non-zero divisor graph, followed by an illustration example. The subsequent outcomes of our investigation presented in the graph.

Definition 2.1: Let R be a ring and I be a proper ideal for R . The ideal-based non-zero divisor graph, denoted by $\emptyset_{I}$ ( *R*), has vertex set *V*( $\emptyset_{I}$ ( *R*)) = R \ { I,1,−1 }. Moreover, two different vertices δ and γ in *V*( $\emptyset_{I}$ ( *R*)) are adjacent if and only if either δγ ∉ I or γδ ∉ I.

**Figure 1.**
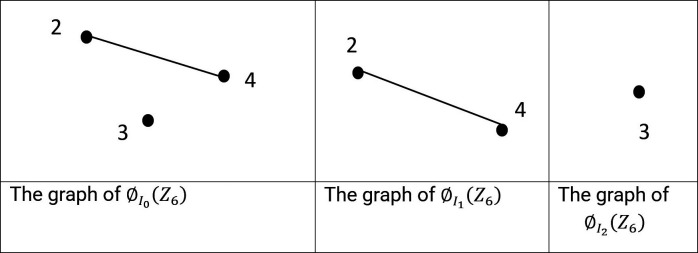
Example for ∅ _*I*_( *R*).

Example 2.2: Let *R* = $Z_{6}$ and let $I_{0}=\left\{0\right\},I_{1}=\left\{0,2,4\;\right\},I_{2}=\left\{0,3\right\}$ . The following Figure 1 are used to illustrate the graph.

Lemma 2.3: If there is an invertible element α in $V\left(\emptyset_{I}\left(R\right)\right)$ , then for any proper ideal I of a ring R , the graph $\emptyset_{I}\left(R\right)$ is connected.

Proof: Because α is invertible then there is β∈R , such that αβ=βα=1 . Let $\gamma \;\in \;V\;(\left(\emptyset_{I}\left(R\right)\right)=R\backslash \left\{I,1,-1\right\}\;$ , if αγ∈Iandγα∈I , then we have βαγ∈Iandγαβ∈I , which is a contradiction. Thus $\emptyset_{I}\left(R\right)$ is connected.

Definition 2.4: ^13^ ϕ(n) denotes the number of positive integers that are co-prime to n , where n≥1.

Theorem 2.5: ^13^If the integer n> 1 has prime factorization $n={P_{1}}^{S_{1}}{P_{2}}^{S_{2}}\ldots {P_{r}}^{S_{r}}\;$ , then $\phi \left(n\right)=\prod_{i=1}^{r}\left({P_{i}}^{S_{i}}-{P_{i}}^{S_{i-1}}\right)$ . ϕ(n) is the number of invertible elements in the integer ring of module n , which is noteworthy.

Lemma 2.6: For *n* > 6, ϕ(n)> 3.

Proof: We shall partition the proof into two distinct cases.

- **Case 1:** *n* is a prime number then ϕ(n) = n-1 > 3.
- **Case 2:** if $n={P_{1}}^{S_{1}}{P_{2}}^{S_{2}}\ldots {P_{r}}^{S_{r}}$ (composite), where $P_{1}$ , $P_{2}$ , …, $P_{r}$ represent *r* different prime numbers. Thus, $\phi \left(n\right)=\prod_{i=1}^{r}\left({P_{i}}^{S_{i}}-{P_{i}}^{S_{i-1}}\right)$ = 3 if and only if j exists such that $\left({P_{j}}^{S_{j}}-{P_{j}}^{S_{j-1}}\right)$ = 3 and $\prod_{i=1\;i\neq j}^{r}\left({P_{i}}^{S_{i}}-{P_{i}}^{S_{i-1}}\right)$ = 1, this is impossible when *n* > 6.

Theorem 2.7: ^9^ The non-zero divisor graph $\emptyset_{0}$ ( $Z_{n}$ ) is connected if and only if *n* ∉ {1, 2, 3, 6}.

## 3. Main results

Connectivity of the graph $\emptyset_{I}$ ( $Z_{n}$ ) is examined in this section for two different scenarios: *n* ≤ 9 and *n* ≥ 10. With the help of examples, we further explore the idea of a cut-set and how it is calculated for graph $\emptyset_{I}$ ( Zn ). For n ∈ {1,2,3,6,8}, the Table 1 below provides information about the ideal-based non-zero divisor graph of $Z_{n}.$

Lemma 3.1: For any prime number n∉{2,3} , $\emptyset_{I}$ ( $Z_{n}$ ) represents a completely connected graph.

Proof: Given that n is a prime number, the only proper ideal for *Z*_*n*_ is the zero ideal. According to Theorem 2.7, $\emptyset_{I}$ ( $Z_{n}$ ) is connected. Assume that $\gamma ,\beta \;\in \;V\left(\emptyset_{I}\left(Z_{n}\right)\right)$ with γβ=0 ; therefore, either γ=0orβ=0 , leading to a contradiction. This indicated that the graph was complete.

Lemma 3.2: For any prime number p≥2 , the graph $\emptyset_{I}$ ( $Z_{P^{2}}$ ) is connected.

Proof: The proper ideal of the ring $Z_{P^{2}}$ are I={0} and J=⟨p⟩ which is maximal ideal of $Z_{P^{2}}$ .

• **Case 1:** $\emptyset_{I}$ ( $Z_{P^{2}}$ ) is connected by Theorem 2.7
• **Case 2:** Assume that $\emptyset_{J}$ ( $Z_{P^{2}}$ ) disconnected graph, there exist γ,δ in different connected component meaning: There is no path between γandδ equivalently, for all α ∈ *V*( $\emptyset_{J}$ ( $Z_{P^{2}}$ )) = $\;Z_{P^{2}}\backslash \left\{J,1,-1\right\}$ either αγ∈Jorαδ∈J , consider the ideal K generated by J∪⟨γ⟩ and since γ∉J , then J⊊K , contradiction.

For the integer module $n,Z_{n}=\left\{1,2,\ldots ,n-1\right\}$ and the ideal $I=dZ_{n}=\left\{0,d,2d,\ldots ,\left(\left(n/d\right)-1\right)d\right\}$ for some integer d\n . Since $V\;\left(\emptyset_{I}\left(\mathrm{Zn}\right)\right)=Z_{n}\backslash \left\{I,1,-1\right\}$ , we can now develop a method to calculate the order of the graph $\;\emptyset_{I}$ ( **Z _n_**) as following: $\left|V\;\left(\emptyset_{I}\left(Z_{n}\right)\right)\right|=n-\frac{n}{d}-2$ , where $\left|I\right|=\frac{n}{d}$ .

Lemma 3.3: Let n be an odd, non-prime number then, the non-zero ideal of **Z _n_** with the greatest order is the ideal generated by the prime number p\n , where $\;p=\sqrt{r^{2}+n}-r$ , for some integer r such that $\sqrt{r^{2}+n}\;\in \;Z$ .

Proof: If n=2k+1 is odd number (not prime), then the ideal has the largest of order is the ideal generated by prime number that divides n, we will provide several cases and present a general formula to determine the largest prime number p that divides n. we know that $\left|V\;\left(\emptyset_{I}\left(\mathrm{Zn}\right)\right)\right|=n-\frac{n}{p}-2$ , where $\left|I\right|=\frac{n}{p}$ . Therefor if p\(n=2k+1) , then $k=\frac{p-1}{2},\left(\frac{p-1}{2}\right)p,\left(\frac{p-1}{2}\right)p+p,\left(\frac{p-1}{2}\right)p+2p,\left(\frac{p-1}{2}\right)p+3p,\ldots$ . Then, the general formula for p is $p=\sqrt{r^{2}+n}-r$ for some integer r that makes $\sqrt{r^{2}+n}\;\in \;Z$ .

Example 3.4: If n=15 take r=1 then p=3 is the greatest order of ideal I such that $\left|V\;\left(\emptyset_{I}\left(\mathrm{Zn}\right)\right)\right|$ = $\;15-\frac{15}{3}-2=8$ .

Theorem 3.5: For any integer n≥10 , the graph $\emptyset_{I}\left(Z_{n}\right)$ is connected for every non-zero proper ideal I of $Z_{n}$ .

Proof: The proof will be divided into two cases.

**Case 1:** *I* is a prime ideal; let γ,δ∈ V ( $\emptyset_{I}$ ( $Z_{n}$ )), with γδ∈ *I*, then either γ∈ *I* or δ∈ *I*, contradiction. Thus $\emptyset_{I}$ ( $Z_{n}$ ) connected (complete).

**Case 2:** I not prime ideal. Therefore, we have the following subcases.

• If n is even number, then the ideal has the largest of order is the ideal generated by 2, $\left|V\;\left(\emptyset_{I}\left(Z_{n}\right)\right)\right|=n-\frac{n}{2}-2=\frac{n}{2}-2$ , that means when n≥10 , then $\left|V\;\left(\emptyset_{I}\left(\mathrm{Zn}\right)\right)\right|$ ≥ 3. By Lemma 2.6, then ϕ(n)> 3, and our result follows immediately by Lemma 2.3.
• If n=2k+1 is an odd number (not prime), then by Lemma 3.3, the ideal with the largest order is the ideal generated by prime number p that divides n, where $\;p=\sqrt{r^{2}+n}-r$ , for some integer *r* such that $\sqrt{r^{2}+n}\;\in \;Z$ . If n>10 and n is an odd number, then the greatest order of ideal *I* guarantees $\left|V\;\left(\emptyset_{I}\left(Z_{n}\right)\right)\right|\geq 3$ , By Lemma 2.6, then ϕ(n)> 3, and our result follows immediately by Lemma 2.3.

Corollary 3.5: For any integer *n* ≥ 10, then $\gamma \left(\emptyset_{I}\left(Z_{n}\right)\right)$ = 1 and $\mathrm{gr}\;\left(\emptyset_{I}\left(Z_{n}\right)\right)=3.$

Proof: For any integer n≥10 , from the previous proof of the theorem 3.3 then $\left|V\;\left(\emptyset_{I}\left(Z_{n}\right)\right)\right|\geq 3$ for any ideal I , and by Lemma 2.6 we have $\gamma (\emptyset_{I}(Z_{n}$ )) = 1 and *gr* ( $\emptyset_{I}\left(Z_{n}\right)$ ) =3 .

**Table 1.** Information about $\emptyset_{I}$ ( $Z_{n}$ ), n ∈ {1,2,3,6,8}.

*n*	Ring $Z_{n}$	Ideal I	Vertex set *V* ( $\emptyset_{I}$ ( $Z_{n}$ ))	Connectivity properties
1	$Z_{1}$	{0}	Empty set	
2	$Z_{2}$	{0}	Empty set	
3	$Z_{3}$	{0}	Empty set	
6	$Z_{6}$	$I_{0}=\left\{0\right\}$ $I_{1}=\left\{0,2,4\;\right\}$ $I_{2}=\left\{0,3\right\}$	*V* ( $\emptyset_{I_{0}}$ ( $\;Z_{6}$ )) = {2,3,4} , *V* ( $\emptyset_{I_{1}}$ ( $\;Z_{6}$ )) = {2,4} , *V* ( $\emptyset_{I_{2}}$ ( $\;Z_{6}$ )) = {3}	$\emptyset_{I_{0}}\;\left(\;Z_{6}\right)$ disconnected $\emptyset_{I_{1}}\left(\;Z_{6}\;\right)\;$ connected $\emptyset_{I_{2}}$ ( $\;Z_{6}$ ) connected
8	$Z_{8}$	$I_{0}=\left\{0\right\}$ $I_{1}=\left\{0,2,4,6\right\}$ $I_{2}=\left\{0,4\right\}$	*V* ( $\emptyset_{I_{0}}$ ( $Z_{8}$ )) = {2,3,4,5,6,} *V* ( $\emptyset_{I_{1}}$ ( $Z_{8}$ )) = {3,5} *V* ( $\emptyset_{I_{2}}$ ( $Z_{8}$ )) = {2,3,5,6}	$\emptyset_{I}\left(Z_{8}\right)$ connected for any ideal *I*

## 4. Cut-set of the graph ∅ _*I*_( *Z*_*n*_)

Definition 4.1: ^14^A cut-set is defined as a set of vertices {β,γ,δ,…} in a connected graph *G*, where *G* can be represented as the union of two subgraphs X and Y , such that

1.
E(X)≠∅andE(Y)≠∅ (the edges set of X and Y are not empty)
2.
E(X)∪E(Y)=E(G) and V(X)∪V(Y)=V(G)

3.
V(X)∩V(Y)={β,γ,δ,…}

4.
X\{β,γ,δ,…}≠∅ and Y\{β,γ,δ,…}≠∅ . Moreover, no proper subset of {β,γ,δ,…} also acts as a cut set for any choice of X and Y .

Here, an example that illustrates the definition of the cut-set in graph

**Figure 2.**
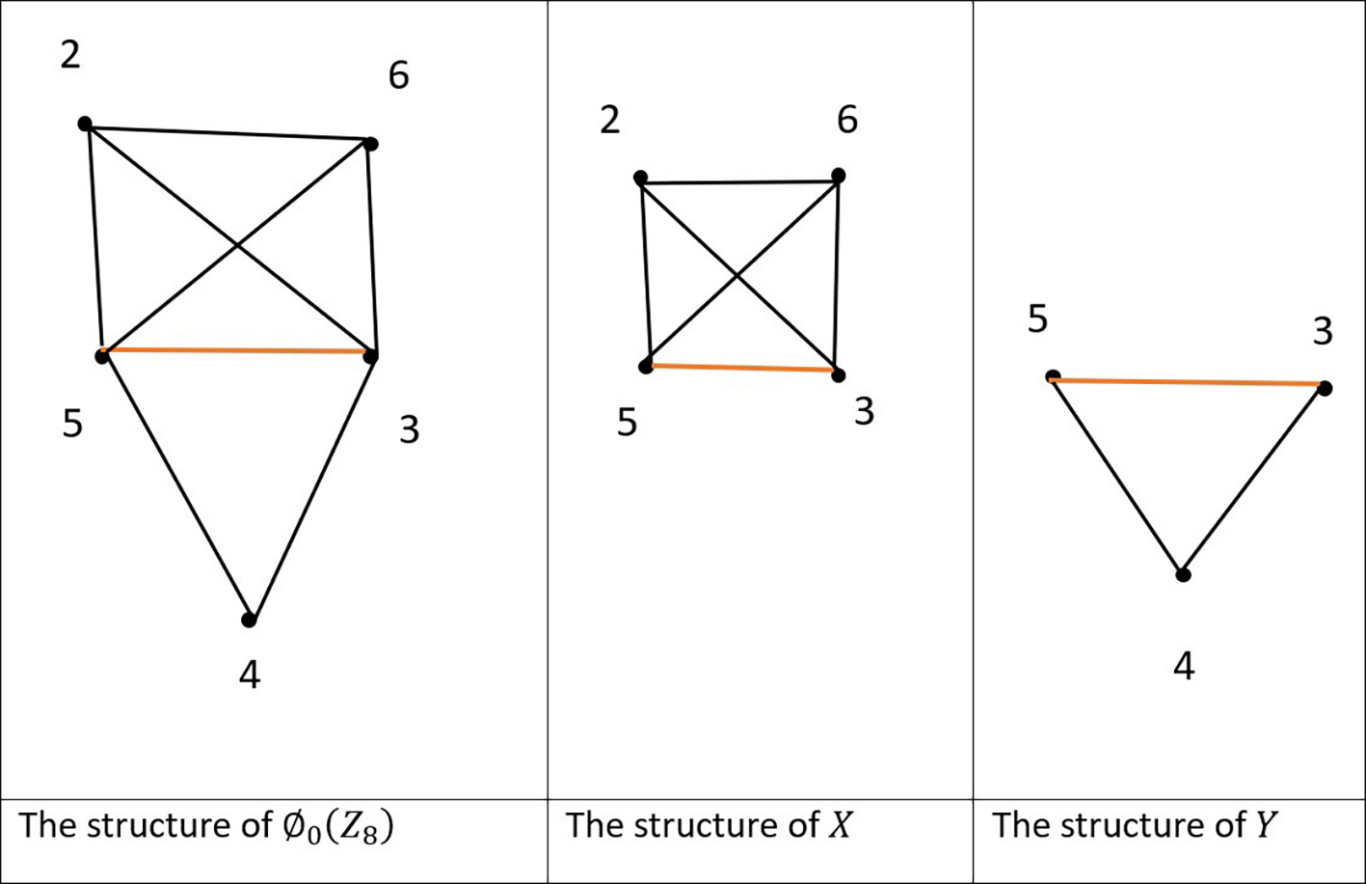
Cut-set of $\emptyset_{0}$ ( *Z*_8_).

Example 4.1: In the graph $\emptyset_{0}\left(Z_{8}\right)$ of ring $Z_{8}$ , the set C={3,5} represents the cut set. As shown Figure 2. $V\;\left(\emptyset_{0}\left(Z_{8}\right)\right)=\left\{2,3,4,5,6\right\}$ .

Proposition 4.2: For every prime number p greater than 2, the cut-set of the non-zero divisor graph $\emptyset_{0}\left(Z_{P^{2}}\right)$ is

$$\left\{\alpha \;\in \;V\left(\emptyset_{0}\left(Z_{P^{2}}\right)\right)\backslash \mathrm{ann}\left(p\right)\right\}.$$

Proof:
$\mathrm{ann}\left(P\right)=\left\{x\;\in \;Z_{P^{2}}|\mathrm{Px}=0\right\}$ = {p,2p,…,(p−1)p} , for x and y in ann(p) , then x=kp and y=hp , for some $\;k,h\;\in \;Z_{P}$ and $\;\mathrm{xy}=\mathrm{kh}P^{2}=0$ , it follows that x is not connected with y . The graph that excludes the vertices of set ann(p) is unconnected. This is minimal because any proper subset of C. C preserves the pathways between zero-divisors via units by leaving some units intact.

**Figure 3.**
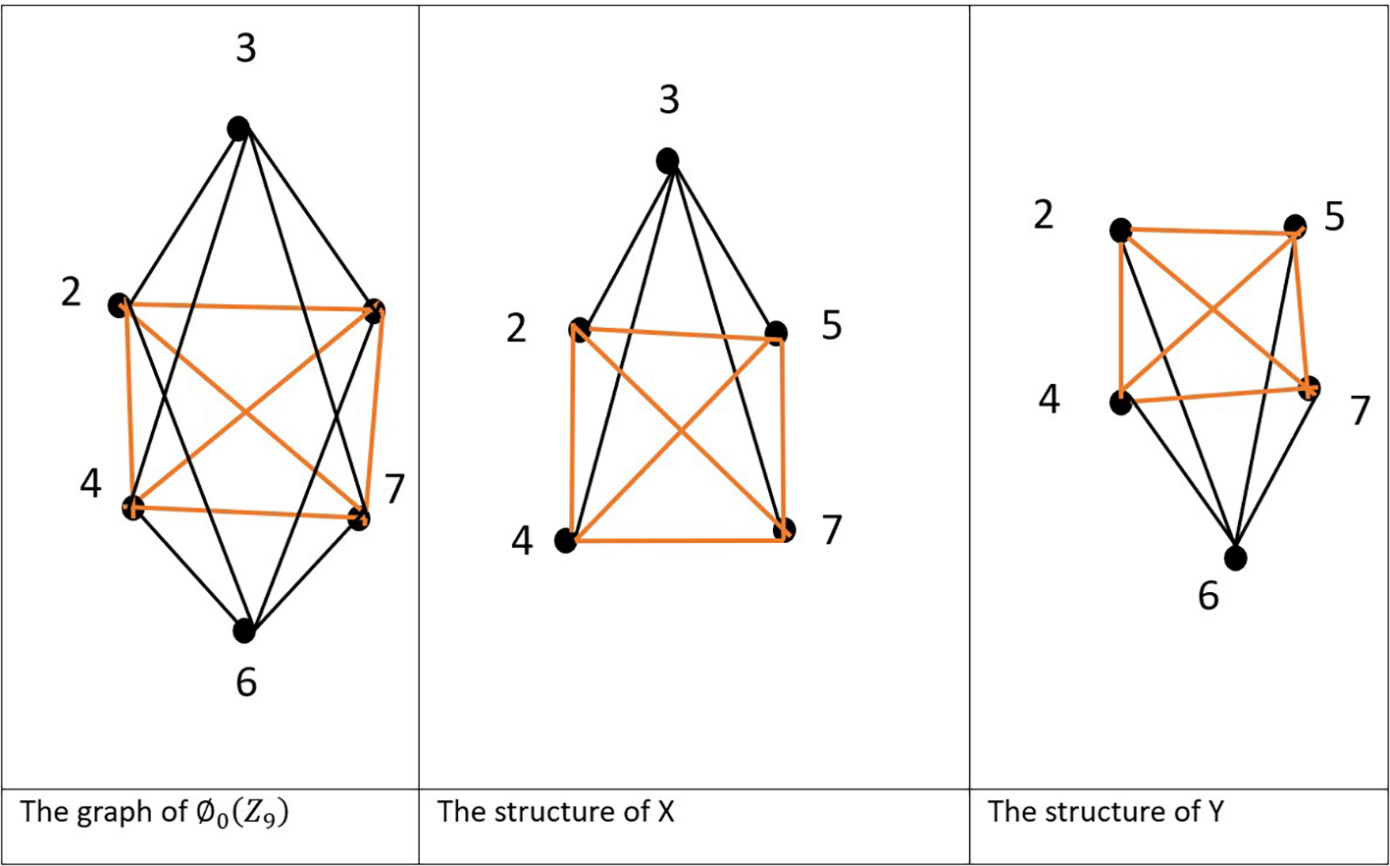
The cut set of $\emptyset_{0}\left(Z_{3^{2}}\right)$ .

Example 4.4: The cut set in the graph $\emptyset_{0}\left(Z_{3^{2}}\right)$ of the ring $Z_{3^{2}}\;$ is the set C = {2,4,5,7}, as illustrated in Figure 3.

Theorem 4.5: Let =x.y , where x≠y and x>y . Then, the Cut-set of $Z_{n}$ , for n>4 , corresponds the set

$$C=P_{n}\cup D_{n}\backslash \left(\left\langle x\right\rangle ⋃\left\langle y\right\rangle \right),$$

where

$$P_{n}=\left\{p\;\in \;v\left((\mathrm{Zn}\;)\right)|\;p\;\text{is prime residues of}\;n\;\right\}$$

$$D_{n}=\left\{d\;\in \;v\left((\mathrm{Zn}\;)\right)|\;d\;\text{is zero divisor of}\;\mathrm{Zn}\right\}$$

Proof: Consider the subgraphs X and Y , which are generated by the ideal ⟨x⟩ and ⟨y⟩ , respectively. Consequently, subgraphs X and Y have no edges between them if γ∈Xandδ∈Y then, δ=0 , Consequently, subgraphs X and *Y* have no edges between them. Now, if $C^{\sim }$ = *C* \ { *a*}, then either $a\;\in \;P_{n}$ , which means that a is invertible, and by Lemma 2.3, a connect all vertices of $\emptyset_{0}\left(Z_{n}\right)$ contradiction, or *a* $\;\in \;D_{n}$ , then there exists path from x to y by a , since if ax≡0(modn) then ax=kn,k∈Z , thus kny=axy , therefore a=xy∈⟨y⟩ , a contradiction.

**Figure 4.**
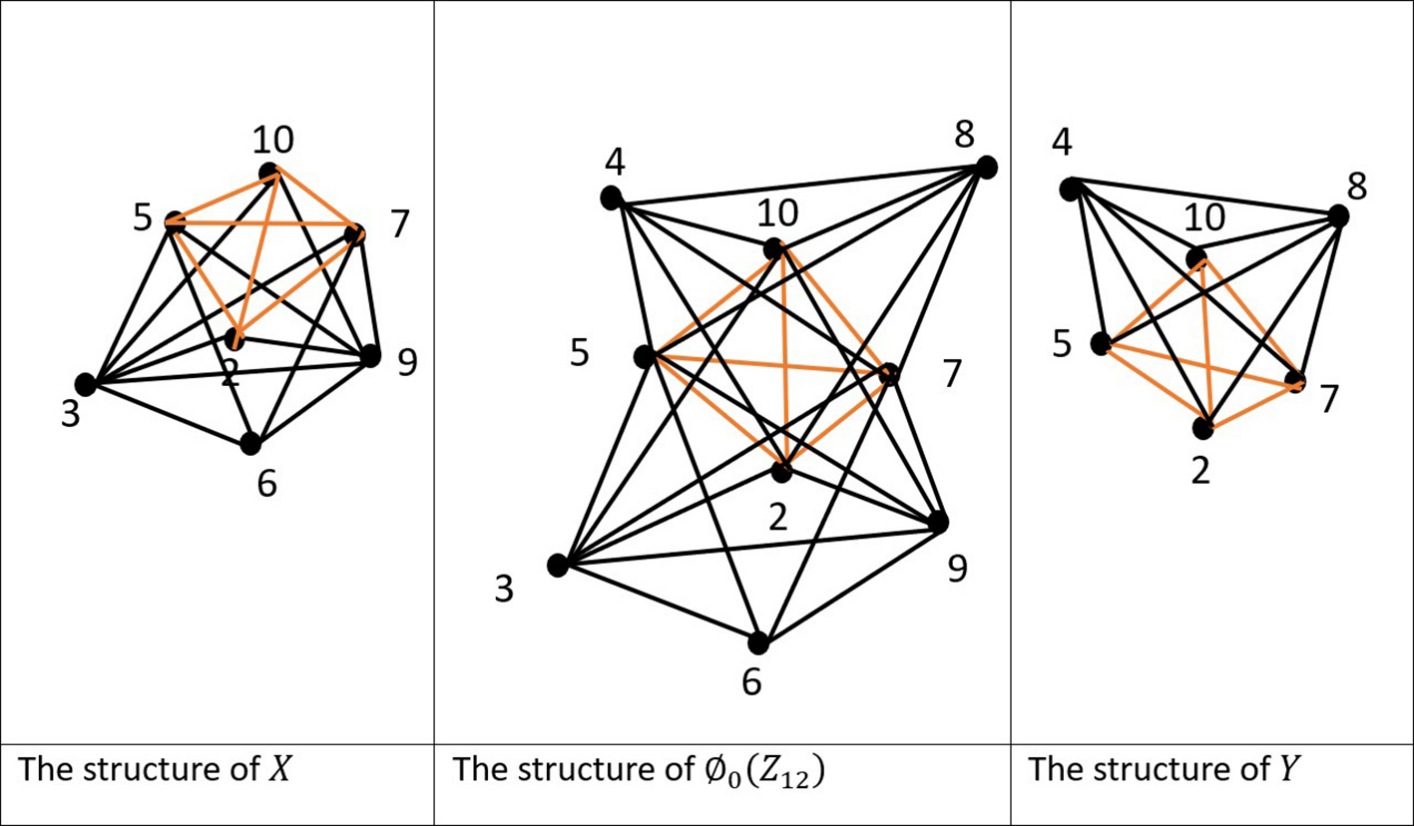
The Cut-set of $\emptyset_{0}\left(Z_{12}\right)$ .

Example 4.3: For the ring $Z_{12}$ , n=3.4 , then the cut set of Zn , for n>4 , corresponds to the set C = $P_{n}\cup D_{n}\backslash \left(\left\langle x\right\rangle ⋃\left\langle y\right\rangle \right)$ = {3,7,210}, as illustrated in the Figure 4, where $P_{n}=\left\{5,7\;\right\}\;\text{and}\;D_{n}=\left\{2,10\right\}$ .

## 5. The degree of a vertex x in $\emptyset_{I}\left(Z_{n}\right)$

Definition 5.1: Let x be a vertex of any graph; the degree of x is represented as deg(x) , and the degree of x is defined as follows: deg(x)=|{y∈V\{x}:d(x,y)=1}| , (d(x,y) length of the short path).

From the above definition, it is clear that deg(x) = $\;\deg(-x)\;in\;\emptyset_{I}(Z_{n})$

Theorem 5.2: Let $R=Z_{n}\;$ be the ring of integers module, and let $\emptyset_{I}$ ( $Z_{n}$ ) denoted the ideal based non-zero divisor graph of R . Suppose I=⟨d⟩ is ideal of, where d\n . for any vertex x∈V ( $\emptyset_{I}$ ( *R*)) and g=gcd(x,d) . Then, the degree of x is given by

$$\deg\left(x\right)=\left\{ \begin{array}{c} \;n-\frac{n}{d}-3\;\text{if}\;g=1\\ n-\frac{n}{g}-2\;\text{if}\;g>1 \end{array}\right.$$

Proof: **Case **1: if g=gcdgcd(x,d)=1 , assume that for all *y* ∈ *V*( $\emptyset_{I}$ ( *R*)), xy∈I=⟨d⟩ , thus d\xy , but gcd(x,d)=1 , therefore, d\y , that means y∈I , a contradiction. Then, by the definition of an ideal-based non-zero divisor, y must be a universal vertex, so $\deg\left(x\right)=\left|V\left(\emptyset_{I}\;\left(R\right)\right)\right|-1=n-2-\left|I\right|-1=n-3-\frac{n}{d}.$

**Case** 2: if g=gcd(x,d)>1 , let x=gl and d=gτ , so we need to exclude the ideal generated by τ , when I want an account for *deg* ( *x*). But $\deg\left(x\right)=n-2-\left|\langle \tau \rangle \right|=n-2-\frac{n}{g}$ .

Example 5.3: Applying Theorem 5.2, we will present a Table 2, illustrating examples of calculating the degree of a vertex in certain $\emptyset_{I}$ ( $Z_{n})$ .

**Table 2.** Calculating the vertex degree in some $\emptyset_{I}$ ( $Z_{n}$ ).

*n*	*I* = ⟨ *d*⟩	*d*	*x*	*gcd*( *x*, *d*) = *g*	*deg*( *x*)
12	I=⟨4⟩	4	5	1	$n-\frac{n}{d}-3=7$
12	I=⟨4⟩	4	10	2	$n-\frac{n}{g}-2=4$
18	I=⟨9⟩	9	15	3	$n-\frac{n}{g}-2=10$
18	I=⟨9⟩	9	10	1	$n-\frac{n}{d}-3=13$
30	I=⟨2⟩	2	7	1	$n-\frac{n}{d}-3=12$
30	I=⟨6⟩	6	21	3	$n-\frac{n}{g}-2=18$

Corollary 5.4: Let $=Z_{P^{2}}$ , P>2 , prime number, for any vertex x ∈ *V* ( $\emptyset_{\left\langle P\right\rangle }$ ( *R*)). The degree of x is then given by $deg(x)=P^{2}-P-3$ .

Corollary 5.5: Let $=Z_{2P}$ , P>2 , prime number, for any vertex *x* ∈ *V* ( $\emptyset_{I}$ ( *R*)), For any non-zero ideal of *R*, then

1.
deg(x)=P−3ifx ∈ *V* ( $\emptyset_{\left\langle 2\right\rangle }$ ( *R*))
2.
deg(x)=2P−5ifx ∈ *V* ( $\emptyset_{\left\langle P\right\rangle }$ ( *R*))

Theorem 5.6: Let *R* = $Z_{n}$ be the ring of integers module n , and let $\emptyset_{0}$ ( $Z_{n}$ ) be a non-zero divisor graph of *R*. Then, for any vertex $x\;\in \;V\;\left(\emptyset_{0}\;\left(R\right)\right),$ set g = deg ( x,n ). Thus, the degree of x is given by:

$$\deg\left(x\right)=\left\{ \begin{array}{ll} n-4 & \text{if}\;g=1\;\\ \;n-g-3 & \text{if}\;x^{2}\neq 0\;\\ n-g-2 & \text{if}\;x^{2}=0\; \end{array}\right.\;$$

Proof: To prove the result, we should consider the following subcases **:**

**Case 1:** if g=gcd(x,n)=1 , assume that for all y ∈ $\;V\;\left(\emptyset_{0}\;\left(R\right)\right)$ , xy=0 , but x is invertible thus, y=0 , which is a contradiction. Therefore $\deg\left(x\right)=\left|V\left(\emptyset_{0}\;\left(R\right)\right)\;\right|-1=n-3-1$ .

**Case 2:** Let $S=\left\{y\;\in \;V\left(\emptyset_{0}\left(R\right)\right):\mathrm{xy}=0\right\}$ , representing the non-neighbors of x , then $S=\mathrm{ann}\left(x\right)\cap V\left(\emptyset_{0}\left(R\right)\right)$ ,which contains exactly g elements if $x^{2}\neq 0$ , and g+1 elements when $x^{2}=0$

Example 5.7: Let $R=Z_{12}$ then the following Table 3, representing the degree of every vertex x of the graph $\emptyset_{0}\left(R\right)$ .

Example 5.8: Let $R=Z_{30}$ then the following Table 4, representing the degree of every vertex x of the graph $\emptyset_{0}\left(R\right)$ .

**Table 3.** The vertex degrees of $\emptyset_{0}$ ( $Z_{12}$ ).

x	gcd(x,12)	$x^{2}\;\mathrm{mod}12$	deg(x)
2,10	2	4	7
3,9	3	9	6
4,8	4	4	5
5,7	1	1	8
6	6	0	4

**Table 4.** The vertex degrees of $\emptyset_{0}$ ( $Z_{30}$ ).

x	g=gcd(x,12)	$x^{2}\;\mathrm{mod}12$	deg(x)
2,28	2	4	25
3,27	3	9	24
4,26	2	16	25
5,25	5	25	22
6,24	6	6	21
7,23	1	19	24
8,22	2	4	25
9,21	3	21	24
10,20	10	10	17
11,19	1	1	26
12,18	6	24	21
13,17	1	19	24
14,16	2	16	25
15	15	15	12

## 6. Topological graph indices

This study calculates the essential topological indices, including the Zagreb and Randić, ^15^ indices, to assess connectedness, symmetry, and complexity. These calculations illustrate the intricate relationship between the ring-theoretic features and graph invariants.

1. **First Zagreb Index:** For any non-zero ideal I of $\;Z_{n}$ , and according to Theorem 5.2, we obtain
$$M_{1}\left(\emptyset_{I}\;\left(Z_{n}\right)\right)=\sum_{x\;\in \;V\left(\emptyset_{I}\;\left(\mathrm{Zn}\right)\right)}{\left(\mathrm{dex}\left(x\right)\right)}^{2}=\left\{ \begin{array}{ll} \sum_{x\;\in \;V\left(\emptyset_{I}\;\left(Z_{n}\right)\right)}{\left(n-\frac{n}{d}-3\right)}^{2} & \text{if}\;g=1\\ \sum_{x\;\in \;V\left(\emptyset_{I}\;\left(Z_{n}\right)\right)}{\left(n-\frac{n}{g}-2\right)}^{2} & \text{if}\;g>1 \end{array}\right.\;$$
  If I=0 , then according to Theorem 5.6, we have
$$\sum_{x\;\in \;V\left(\emptyset_{0}\;\left(Z_{n}\right)\right)}{\left(\mathrm{dex}\left(x\right)\right)}^{2}=\left\{ \begin{array}{ll} \sum_{x\;\in \;V\left(\emptyset_{0}\;\left(Z_{n}\right)\right)}{\left(n-4\right)}^{2} & \text{if}\;g=1\\ \sum_{x\;\in \;V\left(\emptyset_{0}\;\left(Z_{n}\right)\right)}{\left(n-g-3\right)}^{2} & \text{if}\;x^{2}\neq 0\;\\ \sum_{x\;\in \;V\left(\emptyset_{0}\;\left(Z_{n}\right)\right)}{\left(n-g-2\right)}^{2} & \text{if}\;x^{2}=0 \end{array}\right.$$
2. **Second Zagreb Index:** For any non-zero ideal *I* of $Z_{n}$ , let $x,y\;\in \;V\left(\emptyset_{I}\;\left(Z_{n}\right)\right)$ , let $g_{x}=\gcd\left(x,d\right)$ , $g_{y}=\gcd\left(y,d\right)$ and according to Theorem 5.2, we obtain
$$M_{2}\left(\emptyset_{I}\;\left(Z_{n}\right)\right)=\sum_{\mathrm{xy}\in E(\emptyset_{I}\;\left(Z_{n}\right)}\deg\left(x\right)\deg\left(y\right)=\left\{ \begin{array}{ll} \sum_{\mathrm{xy}\in E(\emptyset_{I}\;\left(Z_{n}\right)}{\left(n-\frac{n}{d}-3\right)}^{2} & \text{if}\;g_{x}=1,g_{y}=1\;\\ \sum_{\mathrm{xy}\in E(\emptyset_{I}\;\left(Z_{n}\right)}\left(n-\frac{n}{d}-3\right)\left(n-\frac{n}{g_{y}}-2\right) & \text{if}\;g_{x}=1,g_{y}>1\;\\ \sum_{\mathrm{xy}\in E(\emptyset_{I}\;\left(Z_{n}\right)}\left(n-\frac{n}{g_{x}}-2\right)\left(n-\frac{n}{d}-3\right) & \text{if}\;g_{y}=1,g_{x}>1\;\\ \sum_{\mathrm{xy}\in E(\emptyset_{I}\;\left(Z_{n}\right)}\left(n-\frac{n}{g_{x}}-2\right)\left(n-\frac{n}{g_{y}}-2\right) & \text{if}\;g_{y}>1,g_{x}>1 \end{array}\right.$$
3. **Randić Index**
$$R\left(\emptyset_{I}\;\left(Z_{n}\right)\right)=\sum_{xy\;\in \;E(\emptyset_{I}\;\left(Z_{n}\right)}\frac{1}{\sqrt{\deg\left(x\right)\deg\left(y\right)}}=\left\{ \begin{array}{ll} \sum_{xy\;\in \;E(\emptyset_{I}\;\left(Z_{n}\right)}\frac{1}{\left(n-\frac{n}{d}-3\right)} & \mathrm{if}\;g_{x}=1,g_{y}=1\;\\ \sum_{xy\;\in \;E(\emptyset_{I}\;\left(Z_{n}\right)}\frac{1}{\sqrt{\left(n-\frac{n}{d}-3\right)\left(n-\frac{n}{g_{y}}-2\right)}} & \mathrm{if}\;g_{x}=1,g_{y}>1\\ \sum_{xy\;\in \;E(\emptyset_{I}\;\left(Z_{n}\right)}\frac{1}{\sqrt{\left(n-\frac{n}{g_{x}}-2\right)\left(n-\frac{n}{d}-3\right)}} & \mathrm{if}\;g_{y}=1,g_{x}>1\\ \sum_{xy\;\in \;E(\emptyset_{I}\;\left(Z_{n}\right)}\frac{1}{\sqrt{\left(n-\frac{n}{g_{x}}-2\right)\left(n-\frac{n}{g_{y}}-2\right)}} & \mathrm{if}\;g_{y}>1,g_{x}>1 \end{array}\right.$$

## 7. Conclusion

In this study, we examined the ideal-based non-zero divisor graph $\emptyset_{I}\;\left(Z_{n}\right)$ , emphasizing the relationship between the algebraic structure of $Z_{n}$ and the combinatorial characteristics of its corresponding graph. We developed general formulas for vertex degrees, cut sets, and domination parameters as well as the conditions for graph connectedness. Topological degree-based indices were calculated to measure symmetry and structural complexity. The results show that $\emptyset_{I}\;\left(Z_{n}\right)$ is a useful way to observe and study ideal-related characteristics in modular rings, which connects algebraic and graph-theoretical points of view.

## Data Availability

No data are associated with this article.
