# Supplementary material for: A Study on the Structure of Ideal-Based Non-Zero Divisor Graphs Associated with Z n
Source: F1000Res. 2026 Jan 9;15:44. [Version 1] doi: 10.12688/f1000research.172788.1 (PMC12891953; doi:10.12688/f1000research.172788.1)
Supplement: Peer review history

# Reviewer report: Reviewer response for version 1

**DOI:** 10.5256/f1000research.190544.r449777

**Published:** 2026-02-10

**Authors:** Faisal Al-Sharqi

**Affiliations:** 1 University of Anbar, Ramadi, Iraq

Review on

 A Study on the Structure of Ideal-Based Non-Zero Divisor Graphs Associated with Zn

 Overall Assessment

 This study offers a substantial and systematic exploration of ideal-based non-zero divisor graphs over ∅��(Zn). Its findings—particularly regarding continuity, the nature of segment sets, and the derivation of degree formulas—strengthen the connection between ring theoretical constructs and their graphing representations.

 Technical Quality

 The reasoning is robust and precise. All claims are supported by a logical sequence of results and carefully chosen examples. The work demonstrates extensive expertise in both algebra and graph theory.

 Presentation and Sequence

 The paper is clearly structured, guiding the reader smoothly from basic definitions to key theorems and their applications. Visual aids and tables are effectively used to illustrate key graph properties, making the material more accessible.

 Placement of the Research in Current Studies

 This work fits seamlessly into contemporary algebraic graph theory. By establishing general formulas, conditions, and indicators, this research provides a framework and useful tools for future studies on pattern loops and their graphical constants.

 Final Decision

 The manuscript is clearly written, mathematically accurate, and makes valuable contributions to the field. I support its acceptance for indexing, as it adheres to the conference's scientific standards and will be of interest to researchers in both algebra and graph theory.

Is the work clearly and accurately presented and does it cite the current literature?

Yes

If applicable, is the statistical analysis and its interpretation appropriate?

Not applicable

Are all the source data underlying the results available to ensure full reproducibility?

No source data required

Is the study design appropriate and is the work technically sound?

Yes

Are the conclusions drawn adequately supported by the results?

Yes

Are sufficient details of methods and analysis provided to allow replication by others?

Yes

Reviewer Expertise:

Algebra and its applications

I confirm that I have read this submission and believe that I have an appropriate level of expertise to confirm that it is of an acceptable scientific standard.

# Reviewer report: Reviewer response for version 1

**DOI:** 10.5256/f1000research.190544.r449782

**Published:** 2026-01-27

**Authors:** Nabeel Ezzulddin Arif, Amir Majeed

**Affiliations:** 1 Mathematical Sciences, Tikrit University, Tikrit, Saladin, Iraq; 2 Mathematical Sciences, Sulaimani Polytechnic University (Ringgold ID: 467127), Sulaymaniyah, Kurdistan Region, Iraq

**Review Report: **A Study on the Structure of Ideal-Based Non-

 Zero Divisor Graphs Associated with *Zn*

 This manuscript presents a well-structured and mathematically rigorous study of non-zero divisor schemes and their variants ideal-based in the *Z* *n* ring.

 The results are presented clearly, properly contextualized within the existing literature, and make a valuable contribution to the field. The manuscript's logical flow is sound, and the proofs are valid and thoroughly explained.

 It is recommended that this paper be indexed after the following minor revisions, which mainly concern symbolization, formatting, and presentation.

**Required Revisions:**

 1 -  Lemma 3.3 : The formula  need to rewrite because the largest ideal in Zn is generated by the **smallest prime divisor**, not via this formula.

 2- **Abstract** **:** Justify the text alignment.

**3-** **Figures** **:** Place each figure in the text immediately after its first mention if possible .

 4- **Graph Notation** **(** **Microsoft Equation Editor** **):** **All mathematical symbols, especially those for graphs, should be formatted using Microsoft Equation Editor for consistency and professional presentation.**

 No further mathematical review is required after these editorial changes. The paper is suitable for publication after review.

 Best regards,

 Prof. Dr. Nabeel E. Arif

 Dep. of Mathematics,

 College of Computer Science and Mathematics,

 Tikrit University, Iraq.

 HP: +9647701737803

Is the work clearly and accurately presented and does it cite the current literature?

Yes

If applicable, is the statistical analysis and its interpretation appropriate?

Not applicable

Are all the source data underlying the results available to ensure full reproducibility?

Partly

Is the study design appropriate and is the work technically sound?

Yes

Are the conclusions drawn adequately supported by the results?

Partly

Are sufficient details of methods and analysis provided to allow replication by others?

Yes

Reviewer Expertise:

Graph Theory, Abstract Algebra, Topoligiacl spaces

We confirm that we have read this submission and believe that we have an appropriate level of expertise to confirm that it is of an acceptable scientific standard.
